# Effects of dietary energy and crude protein levels on growth performance, blood profiles, and nutrient digestibility in weaning pigs

**DOI:** 10.5713/ajas.18.0294

**Published:** 2018-08-27

**Authors:** Lin Hu Fang, Ying Hai Jin, Sung Ho Do, Jin Su Hong, Byung Ock Kim, Tae Hee Han, Yoo Yong Kim

**Affiliations:** 1 School of Agricultural Biotechnology, and Research Institute of Agriculture and Life Sciences, Seoul National University, Seoul 08826, Korea; 2 Department of Animal Science, Yanbian University, Yan Ji, Ji Lin, 133000 China

**Keywords:** Energy, Crude Protein, Growth Performance, Blood Profiles, Nutrient Digestibility, Weaning Pigs

## Abstract

**Objective:**

This experiment was conducted to investigate the effect of reducing dietary metabolic energy (ME) and crude protein (CP) levels on growth performance, blood profiles, and nutrient digestibility in weaning pigs.

**Methods:**

A total of 240 crossbred pigs (Duroc×[Landrace×Yorkshire]) with an average body weight of 8.67±1.13 kg were used for a 6-week feeding trial. Experimental pigs were allotted to a 2×3 factorial arrangement using a randomized complete block design. The first factor was two levels of dietary ME density (low ME level, 13.40 MJ/kg or high ME level, 13.82 MJ/kg) and the second factor was three dietary CP levels based on subdivision of early and late weaning phases (low CP level, 19.7%/16.9%; middle CP level, 21.7%/18.9%; or high CP level, 23.7%/20.9%).

**Results:**

Over the entire experimental period, there were no significant difference in body weight among groups, but a decrease in diet energy level was associated with an increase in average daily feed intake (p = 0.02) and decrease in gain-feed ratio (G:F) ratio (p<0.01). Decreased CP levels in the diet were associated with a linear increase in average daily gain (p< 0.05) and quadratic increase in G:F ratio (p<0.05). In the early weaning period, blood urea nitrogen concentration tended to increase when ME in the diet decreased and decrease when CP level in the diet decreased (p = 0.09, p<0.01, respectively). Total protein concentration tended to increase when CP level was reduced (p = 0.08). In the late weaning period, blood urea nitrogen concentration decreased linearly as CP level decreased (p<0.01). The CP and crude fat digestibility decreased when ME was decreased by 0.42 MJ/kg (p = 0.05, p = 0.01, respectively). The CP digestibility increased linearly as CP level decreased (p = 0.01).

**Conclusion:**

A weaning pig diet containing high ME level (13.82 MJ/kg) and low CP level (19.7%/16.9%) can improve pig growth performance and nutrient digestibility.

## INTRODUCTION

Nutritional concentration of feed is very important in the swine industry as it affects both growth performance and profitability. Lower nutritional concentrations can decrease the growth rate of pigs, while higher concentrations can have a negative effect on the environment and increase production costs [1–3]. After weaning, piglets experience nutritional, physiological, environmental, and social stresses [4], which are linked to low feed intake, poor growth, and incidence of diarrhea [5]. In general, a high feed intake could increase the health of weaning pigs and improve growth performance [6,7].

Energy concentration in feed is an important factor of feed intake, as a high energy level can decrease feed intake, while a lower energy level can decrease the deposition of protein [8]. Deposition and intake of protein and amino acids is related to energy concentration [9]. However, the optimal energy requirements of weaning pigs are difficult to determine accurately [10], because different average daily feed intake (ADFI) and weaning stress. Metabolizable energy (ME) requirements in editions of NRC differ, with an increased in ME from 1998 to 2012 [9,11]. However, other studies were reported that ME levels in diets did not affect growth performance in weaning pigs [12,13]. In general, fat and oil are important energy sources in pig diets [14], but weaning pigs do not utilize fat and oil very efficiently [8].

In addition, recommended total N levels have decreased largely over the years in editions of NRC (1998 to 2012). However, other studies reported that the pigs fed low crude protein (CP) diet, showed better fecal consistency score, improved enteric health and growth performance [15–18]. Decreasing the protein level in feed can decrease the risk of post-weaning diarrhea, as undigested proteins can be utilized by pathogenic bacteria such as enterotoxigenic *Escherichia coli* [19]. Moreover, Jensen et al [20] reported that from birth to 56 days, the digestive enzymes of piglets have low activity for digestion of energy and protein. That means there is much more surplus protein and energy that cannot be digested in diets for piglets than requirement. Other study reported that digestibility of CP at the terminal ileum was from 60% to 80% in weaning pigs [21]. Hence, optimizing energy and protein levels in the weaning pig diet is therefore important.

For these many reasons, this experiment was conducted to investigate the effect of reducing dietary ME and CP levels on growth performance, blood profiles, and nutrient digestibility in weaning pigs.

## MATERIALS AND METHODS

### Experimental animals and management

All experimental procedures involving animals were conducted in accordance with the Animal Experimental Guidelines provided by the Seoul National University Institutional Animal Care and Use Committee (SNUIACUC; SNU-160819-9). A total of 240 crossbred pigs (Duroc×[Landrace×Yorkshire]) with an average body weight (BW) of 8.67±1.13 kg were used for a 6-week feeding trial. Pigs were reared at Seoul National University experimental farm. Weaning pigs were allotted to one of six treatments with five replications with 8 pigs per pen. Four male pigs and four female pigs were assigned to each pen of a weaning facility based on BW, and pigs in each pen were maintained until the end of the experiment. Pigs were randomly allotted to their respective treatments by an experimental animal allotment program [22]. Pigs were reared in weaning (1.54×1.96 m) facilities for 6 weeks. Feed and water were provided *ad libitum* during the entire experimental period by a 4-hole stainless feeder and two nipples installed in each pen. The BW and feed intake were recorded at 0, 3, and 6 weeks to calculate average daily gain (ADG), ADFI, and gain-feed ratio (G:F ratio).

### Experimental design and diet

Experimental pigs were allotted to a 2×3 factorial arrangement using a randomized complete block design. The first factor was two levels of dietary ME density (low ME level, 13.40 MJ/kg or high ME level, 13.82 MJ/kg) and the second factor was three dietary CP levels based on subdivision of weaning phases (low CP level, middle CP level, or high CP level), early weaning phase CP percentages of 23.7%, 21.7%, and 19.7% and late weaning phase percentages of 20.9%, 18.9%, and 16.9%.

Experimental diets were formulated for two phases, namely early weaning phase (0 to 3 weeks) and late weaning phase (4 to 6 weeks). Formulae and chemical compositions of the experimental diet are provided in Table 1, 2.

### Blood analysis

Blood samples were taken from the jugular vein of six randomly selected pigs in each treatment when BW were recorded to measure blood urea nitrogen (BUN), glucose, albumin, and total protein after a 3 hours fasting. All blood samples were collected in serum tubes (SST II Advance, BD Vacutainer, Becton Dickinson, Plymouth, UK). Collected blood samples were centrifuged for 15 min at 3,000 rpm at 4°C (5810R, Eppendorf, Hamburg, Germany). Serum was carefully transferred to 1.5 mL plastic tubes and stored at −20°C until analysis. Total BUN (kinetic UV assay, Roche, Mannheim, Germany), albumin (Cobas 8000, Roche, Germany), and glucose (enzymatic kinetic assay, Roche, Germany) concentrations were analyzed using a blood analyzer. Total protein concentration was measured by a kinetic colorimetry assay using a blood analyzer (Modular Analytics, PE, Roche, Germany).

### Digestibility trial

A digestibility trial was conducted using a completely randomized design with three replicates. A total of 18 weaning barrows ([Yorkshire×Landrace]×Duroc) with an average BW of 13.94± 1.64 kg were individually allotted to an individual metabolic crate (0.4×0.8×0.9 m). A 5-day adaptation period was followed by 5 days of data collection.

The daily feed allowance required to provide 2.6 times the maintenance requirements for ME [106 kcal ME/kg (0.44 MJ/kg) of BW^0.75^] was calculated [9,23]. Experimental diets were provided during the late weaning phase at 07:00 and 19:00. At the first and last feeding, 1% ferric oxide and chromium oxide were added to the experimental diet. Water was provided *ad libitum*. Excreta and urine were collected daily and preserved at −20°C until later analysis. When collection was finished, the excreta were dried in an air-forced drying oven at 60°C for 96 h, and ground into 5 mm particles in a Wiley mill for chemical analysis.

### Statistical analyses

All collected data were analyzed using least squares mean comparisons and evaluated using the general linear model procedure implemented in the statistical software package SAS (SAS Institute Inc., Cary, NC, USA). Every pen was considered one unit in the feeding trial, and individual pigs were the experimental units in the digestibility trial and when assessing blood profiles. Orthogonal polynomial contrasts were used to detect linear and quadratic responses to CP levels when the significance of CP was evaluated. Differences were declared significant at p<0.05 or highly significant at p<0.01, while p≥ 0.05 and p<0.10 was considered to indicate a trend in the data.

## RESULTS AND DISCUSSION

### Growth performance

The effect of dietary energy and CP levels on growth performance are presented in Table 3. In the early weaning period, ADG (linear, p<0.01) and ADFI (linear, p<0.05) increased when diet CP level decreased, and ADFI increased when dietary ME decreased (p = 0.04). In the late weaning period, ADFI (p = 0.03) increased and the G:F ratio (p<0.01) decreased when ME level was decreased by 0.42 MJ/kg. There were no significant differences in BW over the experimental period, but decreased ME levels were associated with an increase in ADFI (p = 0.02) and a decrease in G:F ratio (p<0.01). A decrease in CP level resulted in a linear increase in ADG (p<0.05) and a quadratic increase in G:F ratio (p<0.05). Therefore, decreasing the ME level by 0.42 MJ/kg did not affect growth performance, and reducing the CP level by 4% improved ADG and the G:F ratio.

The effect of dietary energy density on weaning pig performance is a topic of debate among researchers. Tokach et al [24] and Hastad et al [25] reported that decreasing dietary energy concentration had no effect on growth. Ribeiro et al [12] reported that reducing dietary ME from 3,700 kcal/kg to 3,250 kcal/kg (15.49 MJ/kg to 13.61MJ/kg) had no influence on growth performance. Beaulieu et al [10] reported that decreasing the digestible energy concentration from 3,650 kcal/kg to 3,350 kcal/kg (15.28 MJ/kg to 14.03MJ/kg) in the weaning pig diet increased BW, ADG, and ADFI linearly, while the G:F ratio decreased linearly.

In this study, reducing the soybean meal (SBM) level to de crease CP level improved growth performance over the whole experiment period. A similar result for ADG and G:F ratio was reported by Li et al [26,27]. Le Bellego and Noblet [28] described a reduction in N excretion and an increase in feed intake when CP level was reduced from 22.4% to 20.4%. In other studies, a lower CP level had no negative effects on growth performance [29,30]. Hermes et al [29] reported that decreasing the CP level from 20% to 16% in the weaning pig (9 to 18 kg) diet had no effect on growth performance after weaning for 3 weeks. Nyachoti et al [30] reported a significant reduction in ADG and ADFI for diets containing a CP level of 19% or less.

### Blood profiles

Blood profiles during the feeding trial are presented in Table 4. The BUN concentration during the early weaning period tended to increase when ME decreased and decrease when CP decreased (p = 0.09, p<0.01, respectively). Total protein concentration tended to increase when CP levels decreased (p = 0.08) in early weaning phase. In the late weaning period, BUN concentration decreased linearly as CP level decreased (p<0.01).

The BUN concentration is an indicator of protein utilization and affects amino acid balance and N-intake [31,32]. Increased energy intake increases protein deposition [33]. This explains why in this study, BUN concentration increased when dietary ME level decreased. BUN concentration decreased when CP level decreased because of optimal amino acid balance and less N-intake. Jeaurond et al [1] and Heo et al [34] reported consistent results with this study, BUN and plasma urea nitrogen concentration decreased when CP level in the weaning pig diet was reduced.

Blood total protein level was increased when pig protein utilization increased [35]. In this study, blood total protein level tended to increase when dietary CP level decreased in weaning pig diet. Also, ADG was increased when dietary CP level deceased. Results in this study indicate that low CP level (19.7%) in the early weaning pig diet is an optimal CP level.

### Nutrient digestibility

Differences in dietary energy and CP levels in the weaning pig diet resulted in significant differences in protein digestibility (Table 5). The CP and crude fat (p = 0.05, p = 0.01, respectively) digestibility decreased when the ME was decreased by 0.42 MJ/kg. The CP digestibility increased in a linear manner when the CP level in the diet decreased (p = 0.01).

This experiment decreased dietary ME concentration by reducing the soy oil content of the feed. Consequently, crude fat digestibility decreased by 3% to 4% when ME in the diet was reduced to 0.42 MJ/kg. Energy digestibility are affected by the type and proportion of ingredients as well as energy source in the diet [10]. Nam and Aherne [36] and the NRC [9] reported that total energy intake could be similar despite different feed intakes because of variations in the energy density of the diet. The ability to digest fat is limited in pigs for the first 35 days of life, but increases after 42 days [10]. Fat digestibility in this experiment results obtained for the late weaning period are in agreement with those of Tokach et al [24] and Beaulieu et al [10].

Lawrence et al [37] and Smith et al [38] reported that dietary energy level affected various nutritional responses. Energy intake is known to influence protein deposition [39]. Lawrence et al [37] and Beaulieu et al [10] reported that the amount of energy in the feed could change N-retention to increase protein digestibility. The results in this study are similar to those if Van Lunen and Cole [40] that decreasing energy density could reduce protein and lipid gain.

The main determinants of protein digestibility are the level and balance of essential amino acids and the animal’s requirement [41]. In this study, lysine %, methionine %, and threonine % were the same for different CP treatments. In other words, although CP levels in the diet were reduced, but the limiting amino acid content was not. In this study, CP digestibility increased when dietary CP levels were decreased. Reducing the CP level without changing the levels of limiting amino acids can result in a more optimal amino acid composition than a high CP level.

## CONCLUSION

Decreasing the energy content of the weaning pig diet by 0.42 MJ/kg (13.82 MJ/kg to 13.40 MJ/kg) had no effects on growth performance, but decreased fat and protein digestibility. In addition, reducing CP level by 4% improved growth performance and the digestibility of proteins, and decreased the BUN level.

A weaning pig diet containing high ME level (13.82 MJ/kg) and low CP level (19.7%/16.9%) can improve pig growth performance and nutrient digestibility.

## Figures and Tables

**Table 1:** Formulae and chemical compositions of the diets of early weaning pigs

Items	Low energy1)			High energy1)		
	
	Low CP (%)2)	Middle CP (%)2)	High CP (%)2)	Low CP (%)2)	Middle CP (%)2)	High CP (%)2)
Ingredient (%)						
Corn	24.80	20.17	15.61	22.74	18.02	13.29
Soybean meal	21.82	27.36	32.84	22.37	27.82	33.27
Barley	33.48	32.76	31.96	32.88	32.27	31.67
Whey powder	8.00	8.00	8.00	8.00	8.00	8.00
Lactose	4.00	4.00	4.00	4.00	4.00	4.00
Soypeptide	4.54	4.74	5.00	4.46	4.72	5.00
Soy-oil	0.00	0.00	0.00	2.18	2.18	2.18
Mono-dicalcium phosphate	1.25	1.14	1.05	1.27	1.17	1.07
Limestone	0.93	0.95	0.97	0.92	0.94	0.96
L-lysine-HCl, 78%	0.39	0.20	0.01	0.39	0.20	0.00
DL-methionine, 80%	0.07	0.04	0.01	0.07	0.04	0.01
L-threonine, 99%	0.17	0.09	0.00	0.17	0.09	0.00
Vit. Mix3)	0.10	0.10	0.10	0.10	0.10	0.10
Min. Mix4)	0.10	0.10	0.10	0.10	0.10	0.10
Salt	0.30	0.30	0.30	0.30	0.30	0.30
ZnO	0.05	0.05	0.05	0.05	0.05	0.05
Chemical composition (calculated value)						
Metabolic energy (MJ/kg)	13.40	13.40	13.40	13.82	13.82	13.82
Crude protein (%)	19.70	21.70	23.70	19.70	21.70	23.70
Total lysine (%)	1.35	1.35	1.35	1.35	1.35	1.35
Total methionine (%)	0.35	0.35	0.35	0.35	0.35	0.35
Total threonine (%)	0.89	0.89	0.89	0.89	0.89	0.89
Calcium (%)	0.80	0.80	0.80	0.80	0.80	0.80
Total phosphorus (%)	0.65	0.65	0.65	0.65	0.65	0.65
Chemical composition (analyzed value)						
Crude protein (%)	20.46	21.76	23.85	19.39	21.24	22.77
Crude fat (%)	1.26	1.71	1.96	3.92	3.96	3.37
Crude ash (%)	6.41	6.31	6.17	6.23	6.48	6.77

CP, crude protein; ME, metabolic energy.

1) Low energy level, ME (13.40 MJ/kg); high energy level, ME (13.82 MJ/kg).

2) Low CP level: early weaning phase CP percentages of 19.7% and late weaning phase percentages of 16.9%; middle CP level: early weaning phase CP percentages of 21.7%, and late weaning phase percentages of 18.9%; high CP level: early weaning phase CP percentages of 23.7%, and late weaning phase percentages of 20.9%.

3) Provided per kg of diet. Vitamins per kg of complete diet: vitamin A, 8,000 IU; vitamin D_3_, 1,800 IU; vitamin E, 60 IU; vitamin K_3_, 2 mg; thiamine, 2.00 mg; riboflavin, 7.0 mg; pantothenic acid, 25 mg; niacin, 27 mg; pyridoxine, 3 mg; d-biotin, 0.2 mg; folic acid, 1 mg; vitamin B_12_, 0.03 mg.

4) Provided per kg of diet. Minerals per kg of complete diet: Se, 0.3 mg; I, 1 mg; Mn, 51.6 mg; Cu, 105 mg; Fe, 150 mg; Zn, 72 mg; Co, 0.5 mg.

**Table 2:** Formulae and chemical compositions of diets in late weaning pigs

Items	Low energy1)			High energy1)		
	
	Low CP (%)2)	Middle CP (%)2)	High CP (%)2)	Low CP (%)2)	Middle CP (%)2)	High CP (%)2)
Ingredient (%)						
Corn	43.00	38.28	33.49	40.91	36.07	31.19
Soybean meal	21.09	25.48	29.86	21.66	25.94	30.22
Barley	25.65	25.50	25.44	25.04	25.10	25.17
Whey powder	3.00	3.00	3.00	3.00	3.00	3.00
Lactose	3.00	3.00	3.00	3.00	3.00	3.00
Soypeptide	0.81	1.81	2.81	0.75	1.80	2.83
Soy-oil	0.26	0.13	0.00	2.45	2.30	2.17
Mono-dicalcium phosphate	1.22	1.12	1.02	1.25	1.13	1.03
Limestone	0.82	0.84	0.85	0.81	0.83	0.85
L-lysine-HCl, 78%	0.38	0.19	0.00	0.37	0.18	0.00
DL-methionine, 80%	0.07	0.04	0.00	0.06	0.04	0.01
L-threonine, 99%	0.17	0.08	0.00	0.17	0.08	0.00
Vit. Mix3)	0.10	0.10	0.10	0.10	0.10	0.10
Min. Mix4)	0.10	0.10	0.10	0.10	0.10	0.10
Salt	0.30	0.30	0.30	0.30	0.30	0.30
ZnO	0.03	0.03	0.03	0.03	0.03	0.03
Chemical composition (calculated value)						
Metabolic energy (MJ/kg)	13.40	13.40	13.40	13.82	13.82	13.82
Crude protein (%)	16.90	18.90	20.90	16.90	18.90	20.90
Total lysine (%)	1.15	1.15	1.15	1.15	1.15	1.15
Total methionine (%)	0.31	0.31	0.31	0.30	0.31	0.31
Total threonine (%)	0.80	0.80	0.80	0.80	0.80	0.80
Calcium (%)	0.70	0.70	0.70	0.70	0.70	0.70
Total phosphorus (%)	0.60	0.60	0.60	0.60	0.60	0.60
Chemical composition (analyzed value)						
Crude protein (%)	18.64	19.65	22.63	18.26	18.61	20.47
Crude fat (%)	1.95	1.56	1.63	4.26	5.34	4.04
Crude ash (%)	4.91	5.23	5.67	5.01	5.86	5.84

CP, crude protein; ME, metabolic energy.

1) Low energy level, ME (13.40 MJ/kg); high energy level, ME (13.82 MJ/kg).

2) Low CP level: early weaning phase CP percentages of 19.7% and late weaning phase percentages of 16.9%; middle CP level: early weaning phase CP percentages of 21.7%, and late weaning phase percentages of 18.9%; high CP level: early weaning phase CP percentages of 23.7%, and late weaning phase percentages of 20.9%.

3) Provided per kg of diet. Vitamins per kg of complete diets: vitamin A, 8,000 IU; vitamin D_3_, 1,800 IU; vitamin E, 60 IU; vitamin K_3_, 2 mg; thiamine, 2.00 mg; riboflavin, 7.0 mg; pantothenic acid, 25 mg; niacin, 27 mg; pyridoxine, 3 mg; d-biotin, 0.2 mg; folic acid, 1 mg; vitamin B_12_, 0.03 mg.

4) Provided per kg of diet. Minerals per kg of complete diet: Se, 0.3 mg; I, 1 mg; Mn, 51.6 mg; Cu, 105 mg; Fe, 150 mg; Zn, 72 mg; Co, 0.5 mg.

**Table 3:** Effects of dietary energy and crude protein levels on growth performance in weaning pigs

Items	Low energy1)			High energy1)			SEM	p-value		
		
	Low CP (%)2)	Middle CP (%)2)	High CP (%)2)	Low CP (%)2)	Middle CP (%)2)	High CP (%)2)		ME	CP	M×C
Body weight (kg)										
Initial	8.66	8.66	8.67	8.67	8.67	8.67	0.206	0.99	1.00	1.00
3 weeks	13.59	12.23	12.88	13.33	12.23	12.34	0.280	0.65	0.22	0.93
6 weeks	24.28	22.24	23.45	24.52	22.54	22.94	0.406	0.99	0.16	0.90
ADG (g)										
0–3 weeks3)	234^a^	170^c^	201^b^	222^a^	170^c^	175^c^	7.6	0.33	<0.01	0.72
4–6 weeks	509	477	505	533	491	505	7.8	0.41	0.17	0.84
0–6 weeks3)	372	323	352	377	330	340	6.5	0.99	0.01	0.76
ADFI (g)										
0–3 weeks3)	433^a^	362^b^	388^b^	391^b^	335^c^	325^c^	10.6	0.04	0.01	0.84
4–6 weeks	986	970	1,030	924	925	915	16.7	0.03	0.82	0.66
0–6 weeks	709	666	704	658	630	620	11.9	0.02	0.44	0.67
G:F ratio										
0–3 weeks	0.542	0.462	0.528	0.569	0.508	0.548	0.0148	0.31	0.15	0.93
4–6 weeks	0.518^ab^	0.498^b^	0.490^b^	0.576^a^	0.532^ab^	0.554^a^	0.0084	<0.01	0.17	0.65
0–6 weeks4)	0.524^b^	0.488^c^	0.502^bc^	0.575^a^	0.526^b^	0.549^ab^	0.0080	<0.01	0.04	0.91

CP, crude protein; SEM, standard error of the mean; ME, metabolic energy; ADG, average daily gain; ADFI, average daily feed intake; G:F, gain-feed ratio.

1) Low energy level, ME (13.40 MJ/kg); high energy level, ME (13.82 MJ/kg).

2) Low CP level: early weaning phase CP percentages of 19.7% and late weaning phase percentages of 16.9%; middle CP level: early weaning phase CP percentages of 21.7%, and late weaning phase percentages of 18.9%; high CP level: early weaning phase CP percentages of 23.7%, and late weaning phase percentages of 20.9%.

3) Linear response (p<0.05) to dietary CP levels when a significant CP effect was detected.

4) Quadratic response (p<0.05) to dietary CP level when a significant CP effect was detected.

abc Means with different superscripts in the same row differ significantly (p<0.05).

**Table 4:** Effects of dietary energy and crude protein levels on blood profiles in weaning pigs

Items	Low energy1)			High energy1)			SEM	p-valve		
		
	Low CP (%)2)	Middle CP (%)2)	High CP (%)2)	Low CP (%)2)	Middle CP (%)2)	High CP (%)2)		ME	CP	M×C
Albumin (g/L)										
Initial	39.83	-	-	-	-					
3 weeks	30.40	31.80	30.00	30.20	31.00	30.20	0.543	0.82	0.63	0.94
6 weeks	31.40^b^	30.00^b^	34.20^a^	30.40^b^	32.60^ab^	25.80^c^	0.796	0.12	0.94	0.01
BUN (mmol/L)										
Initial	3.14	-	-	-	-					
3 weeks3)	3.93^bc^	4.92^b^	5.51^a^	2.37^c^	4.89^b^	4.85^b^	0.271	0.09	<0.01	0.35
6 weeks3)	2.75^c^	3.81^b^	5.47^a^	2.11^c^	3.46^b^	4.82^ab^	0.266	0.13	<0.01	0.92
Glucose (mmol/L)										
Initial	6.67	-	-	-	-					
3 weeks	5.11	5.42	4.83	5.29	5.11	5.18	0.112	0.74	0.65	0.49
6 weeks	5.51	5.24	5.59	5.39	5.79	5.25	0.094	0.86	0.92	0.16
Total protein (g/L)										
Initial	53.10	-	-	-	-					
3 weeks	51.84	51.61	49.02	50.81	51.78	46.21	0.758	0.21	0.08	0.60
6 weeks	56.38	54.63	59.39	54.61	55.24	54.63	0.649	0.12	0.38	0.23

CP, crude protein; SEM, standard error of the mean; ME, metabolic energy; BUN, blood urea nitrogen.

1) Low energy level, ME (13.40 MJ/kg); high energy level, ME (13.82 MJ/kg).

2) Low CP level: early weaning phase CP percentages of 19.7% and late weaning phase percentages of 16.9%; middle CP level: early weaning phase CP percentages of 21.7%, and late weaning phase percentages of 18.9%; high CP level: early weaning phase CP percentages of 23.7%, and late weaning phase percentages of 20.9%.

3) Linear response (p<0.05) to dietary CP levels when a significant CP effect was detected.

abc Means with different superscripts in the same row differ significantly (p<0.05).

**Table 5:** Effects of dietary energy and crude protein levels on nutrient digestibility in weaning pigs

Nutrient digestibility (%)	Low energy1)			High energy1)			SEM	p-valve		
		
	Low CP (%)2)	Middle CP (%)2)	High CP (%)2)	Low CP (%)2)	Middle CP (%)2)	High CP (%)2)		ME	CP	M×C
Dry matter	88.01	87.87	88.24	89.12	88.66	88.51	0.727	0.88	0.81	0.91
Crude protein3)	87.81^ab^	85.07^b^	84.24^b^	89.41^a^	85.21^b^	85.11^b^	1.459	0.05	0.01	0.21
Crude ash	71.19	72.31	71.21	70.90	71.64	73.14	0.773	0.21	0.27	0.31
Crude fat	74.51^b^	73.41^b^	72.87^b^	78.74^a^	76.15^ab^	75.71^ab^	1.181	0.01	0.18	0.87

CP, crude protein; SEM, standard error of the mean; ME, metabolic energy.

1) Low energy level, ME (13.40 MJ/kg); high energy level, ME (13.82 MJ/kg).

2) Low CP level: early weaning phase CP percentages of 19.7% and late weaning phase percentages of 16.9%; middle CP level: early weaning phase CP percentages of 21.7%, and late weaning phase percentages of 18.9%; high CP level: early weaning phase CP percentages of 23.7%, and late weaning phase percentages of 20.9%.

3) Linear response (p<0.05) to dietary CP levels when a significant CP effect was detected.

ab Means with different superscripts in the same row differ significantly (p<0.05).
